# Improving network inference algorithms using resampling methods

**DOI:** 10.1186/s12859-018-2402-0

**Published:** 2018-10-12

**Authors:** Sean M Colby, Ryan S McClure, Christopher C Overall, Ryan S Renslow, Jason E McDermott

**Affiliations:** 1 Earth and Biological Sciences Directorate, Pacific Northwest National Laboratory, Richland, Washington, USA; 2 Present Address: Center for Brain Immunology and Glia, University of Virginia, Charlottesville, Virginia, USA

**Keywords:** Gene regulatory network inference, Random subspace method, Resampling, Bootstrapping, Aggregation

## Abstract

**Background:**

Relatively small changes to gene expression data dramatically affect co-expression networks inferred from that data which, in turn, can significantly alter the subsequent biological interpretation. This error propagation is an underappreciated problem that, while hinted at in the literature, has not yet been thoroughly explored. Resampling methods (e.g. bootstrap aggregation, random subspace method) are hypothesized to alleviate variability in network inference methods by minimizing outlier effects and distilling persistent associations in the data. But the efficacy of the approach assumes the generalization from statistical theory holds true in biological network inference applications.

**Results:**

We evaluated the effect of bootstrap aggregation on inferred networks using commonly applied network inference methods in terms of stability, or resilience to perturbations in the underlying expression data, a metric for accuracy, and functional enrichment of edge interactions.

**Conclusion:**

Bootstrap aggregation results in improved stability and, depending on the size of the input dataset, a marginal improvement to accuracy assessed by each method’s ability to link genes in the same functional pathway.

**Electronic supplementary material:**

The online version of this article (10.1186/s12859-018-2402-0) contains supplementary material, which is available to authorized users.

## Background

Little is known about gene regulatory networks, even among the simplest bacteria: *Escherichia coli*. *E. coli* has 4,377 genes and thus almost 10 million possible binary edge interactions. If more complicated interaction motifs (e.g. feed-forward, cascade, fan-in, fan-out) are considered, this number grows to 14 billion for 3-gene interactions, and 15 trillion for 4-gene. In addition, few interactions in *E. coli* have been confirmed through experimentation [1], and even fewer in more complex organisms, such as *S. cerevisiae* [2]. This results in an inability to assess the performance of inferred networks in all but the simplest organisms, and even then with a significantly limited set of known interactions with which to make a comparison. Synthetic networks have been used to circumvent the absence of fully annotated interaction networks, but performance does not necessarily generalize to real world applications [3].

**Fig. 1:**
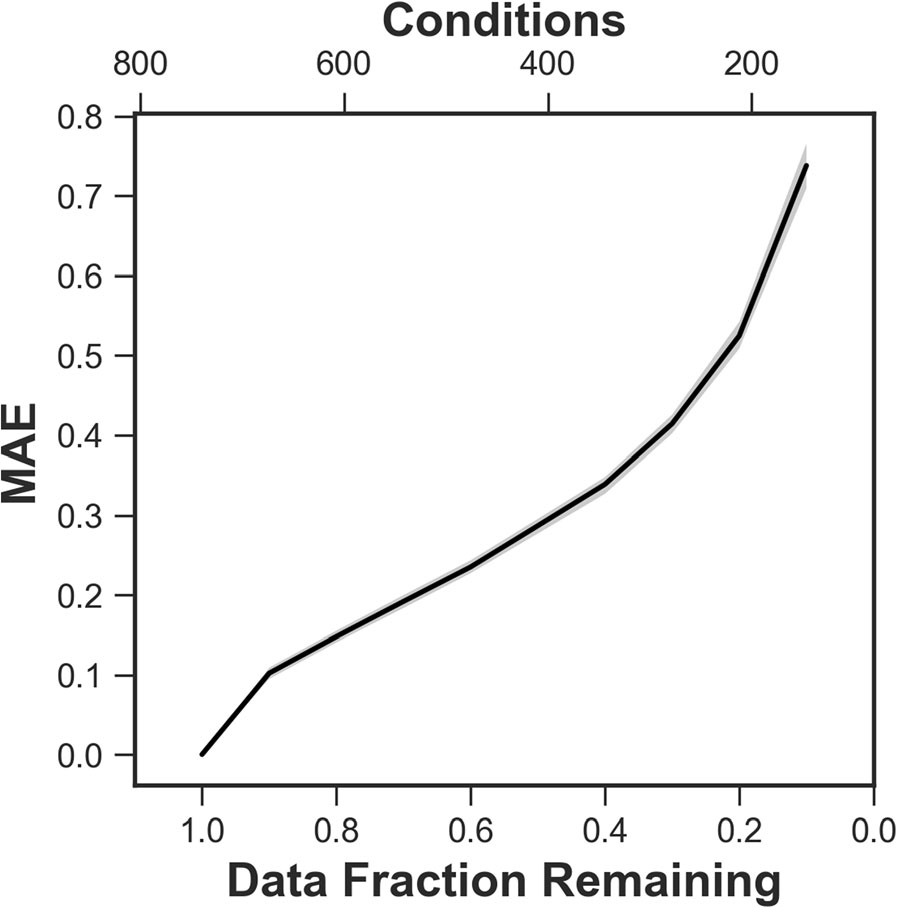
Stability assessment. Stability of *E. coli* network inference by CLR during condition removal, assessed in terms of MAE. Bands indicate a 99% confidence interval constructed from the samples taken at each data fraction

Still, extensive research has been performed to infer such relationships from experimental gene expression data through supervised and unsupervised learning methods. This effort has yielded a number of algorithms and computational techniques to tease apart network interactions. The Dialogue for Reverse Engineering Assessments and Methods (DREAM) challenge aims to evaluate the success of gene regulatory network inference and has used standards for evaluation of network accuracy based on known regulator-target relationships [3]. However, inferred networks contain edges that vary widely in their confidence, and our previous research has shown that even small changes in the input data—removing a few conditions in the expression data set, for example—can result in large changes in the resulting network (Fig. 1). Several studies, our own included, have reported the use of resampling methods in conjunction with network inference to achieve a.

higher level of stability in the resulting networks [4–7], though it remains unclear how effective this approach is for improving.

network inference results, despite success in other applications where “truth” is known [8–11].

Other methods have been developed for statistical aggregation in network inference [12–16]. Filosi et al. 2014 coined several indicators of stability through use of resampling by cross validation (specifically: leave-one-out, k-fold). These stability indicators offer quantitative metrics to assess the resilience of a given method to the presence/absence of conditions, but resampled networks were not aggregated in an attempt to improve predictions [12]. In de Matos Simoes et al. 2012, bootstrap aggregation was applied to the C3NET (Conservative Causal Core network) algorithm, thus termed bootstrapped-C3NET, or BC3NET, demonstrating improvement in terms of F1 score compared to C3NET. Their approach additionally provided a statistical procedure for determining an optimal confidence threshold parameter, a nontrivial selection, using the networks generated during bootstrapping [13]. Guo et al. 2017 employed use of partial correlations (i.e. isolation of a single gene pair at a time), extracting only the most highly correlated relationships as edges in their RLowPC (Relevance Low order Partial Correlation) method [17]. Friedman et al. 1999 applied bootstrapping to yield a successful result, but by resampling genes, not conditions, and applying to small, synthetic datasets [14]. GENIE3 (GEne Network Inference with Ensemble of trees) [15] and ARCANE (Algorithm for the Reconstruction of Accurate Cellular Networks) [16] implicitly perform subspace resampling, but neither evaluate the associated effects explicitly. Our work expands on these previous efforts by focusing on the relationship between the initial set of expression conditions used to infer the network and parameters of the resampling approaches to both stability and accuracy of the resulting networks, applied to real-world datasets.

We evaluate feature bootstrap aggregating as a potential remedy to the observed variability in network inference applications, in terms of both stability (i.e. resistance to variability in inferred relationships) and accuracy, as scored against the standards. We then address the validity of the assumption that stability and accuracy are correlated, as this has come up in network inference problems in which direct evaluation is not possible [4]. Finally, we show that bootstrapping improves both stability and accuracy when inferring networks from datasets, contingent on the underlying inference method and the number of input conditions.

## Results

### Sensitivity to number of conditions

To examine the effects of removing data from the dataset used for network inference we obtained a large set of transcriptomics data representing > 800 conditions (differences in growth conditions, genetic differences, time points, etc.) for *E. coli* used by the DREAM5 competition [3]. We first applied a standard mutual-information based method for network inference, the context-likelihood of relatedness (CLR) algorithm [18], to the entire dataset to generate a network. Sets of individual conditions were removed randomly in successively larger amounts and constituent networks were inferred based on the subset of conditions, 10 times for each step. Mean absolute error (MAE), or the average of the absolute differences between two sets of observations, of each constituent network was calculated relative to the parent network. Figure 1 shows the effect of such perturbations. As we previously observed with a much smaller starting set of data in *Synechoccocus* [4], as more conditions are removed, the resulting constituent networks generated by CLR increasingly differ from the network generated with all conditions (the parent network). Though approaches have been used that purport to address this variability issue, the efficacy of these approaches have not been rigorously evaluated [4, 5, 13, 15, 16].

### Accuracy

To replicate the bootstrapping approach commonly used by us and others, we subsample a specific fraction of the total dataset a number of times and then aggregate the results by averaging edge weights. Using the CLR method as the underlying network inference method, we refer to this approach as BCLR.

**Fig. 2:**
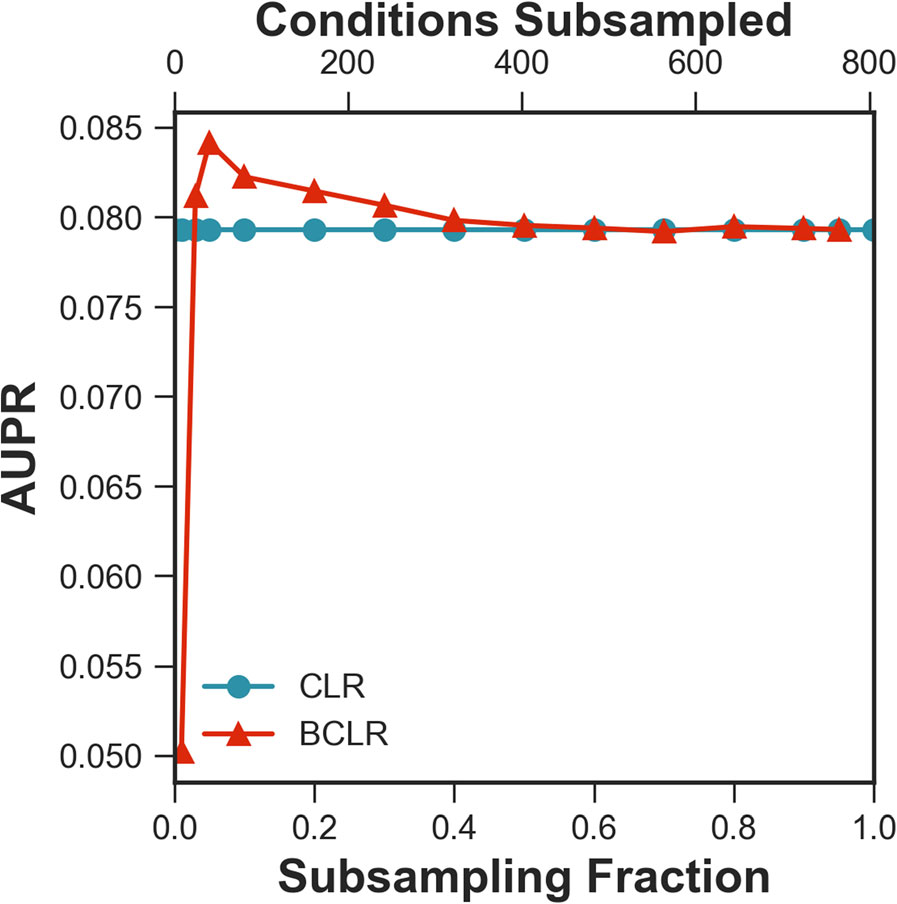
Subsampling fraction sensitivity. A sweep across subsampling fractions is performed to show performance of BCLR on the *E. coli* dataset. The largest difference between methods, at 5% subsampling fraction, was determined significant with *p*-value 4.9E-9

We first examined the accuracy of the BCLR approach on the entire *E. coli* dataset while varying the subsampling fraction used (Fig. 2). We found that 200 iterations were sufficient for BCLR to converge (Additional file 1: Supplemental Results and Additional file 2: Figure S1). We evaluated accuracy as area under the precision-recall (AUPR) curve with known transcription factor-target relationships that were used to evaluate the DREAM5 competition [3]. At a subsampling fraction of 5% (that is, keeping random subsets of 5% of the ~ 800 conditions, or ~ 40 conditions), a performance increase over CLR of ~ 6% (*p*-value: 4.9E-9) is observed, meaning BCLR was able to outperform CLR when subsampling from the full set of conditions.

### Stability

Network inference methods require data from a number of perturbations to be able to draw accurate inferences of real relationships. Based on the premise of bootstrapping, and results reported elsewhere [4, 12, 13], we hypothesized that bootstrapping would improve the stability of the inferred network relative to the parent network inferred with all the conditions. We therefore assessed the stability of BCLR with 5% subsampling fraction over a range of different initial dataset sizes.

**Fig. 3:**
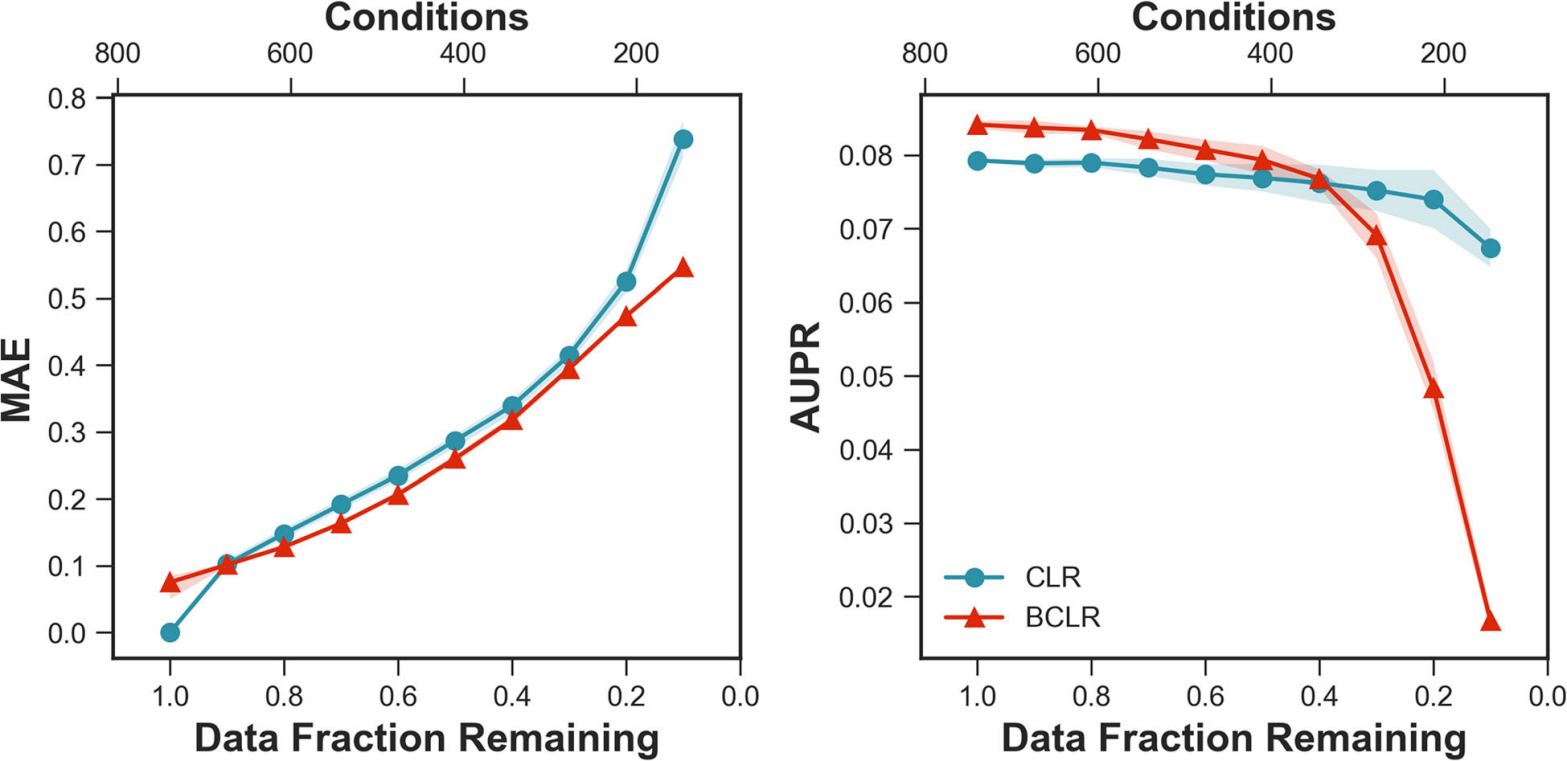
Dataset size sensitivity. Subsampling fraction held constant (5%) and number of conditions are varied to demonstrate stability in terms of MAE (left) and effect on accuracy in terms of AUPR (right) on the *E. coli* dataset. Bands indicate a 99% confidence interval constructed from the samples taken at each data fraction. For MAE, all differences were significant (*p*-value < 0.01) *except* for 0.9 data fraction remaining (*p*-value: 0.77). For AUPR, all differences were significant (*p*-value < 0.01) *except* for 0.4 and 0.5 data fraction remaining (*p*-values of 0.63 and 0.04, respectively)

Figure 3 shows that when considering all the data, the stochastic nature of BCLR with 5% subsampling fraction causes the resulting networks to differ from the parent network (inferred using all the data). However, with smaller numbers of conditions—fewer than about 160—BCLR demonstrates improved stability over CLR, and this is particularly accentuated at small initial dataset sizes.

In terms of accuracy, Fig. 3 demonstrates the effectiveness of BCLR across the majority of condition set sizes. With larger initial dataset sizes BCLR outperforms CLR in its ability to infer correct relationships among transcription factor-gene interactions. For smaller condition sets (i.e. > 50% removed), CLR appears to be more robust to the loss of conditions. However, 40% of the full set is 322 conditions, meaning BCLR with 5% subsampling fraction only “sees” 16 conditions during each iteration.

### Dataset size

**Fig. 4:**
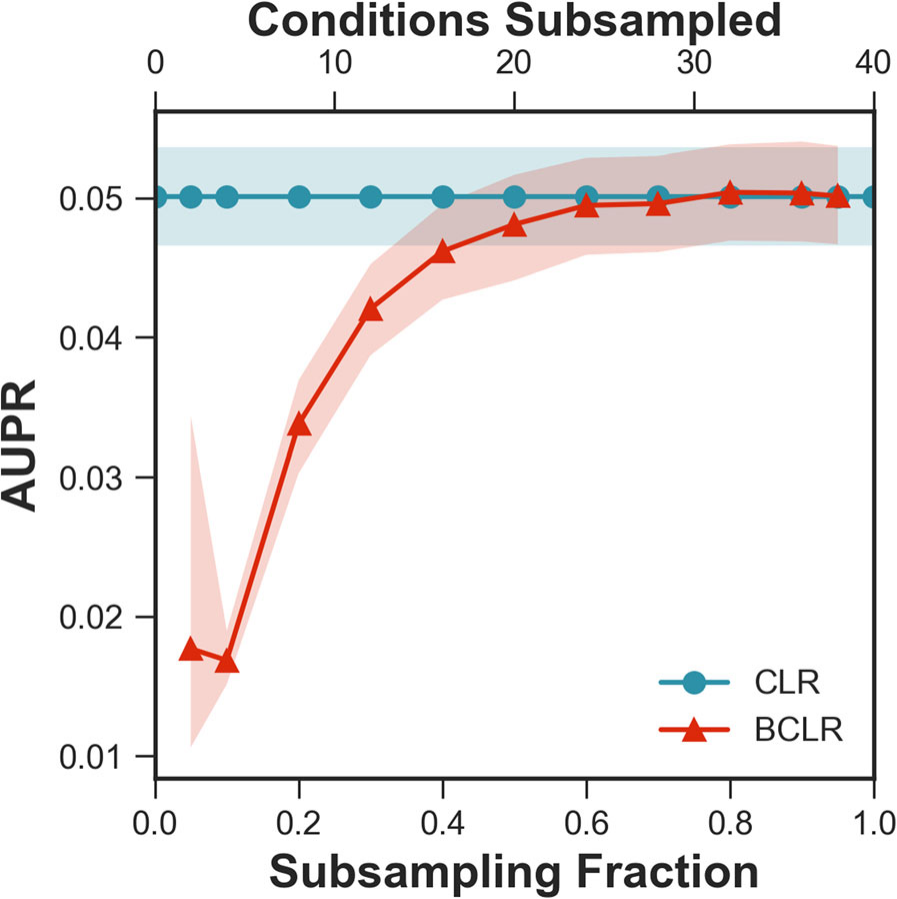
Realistically sized dataset. Number of conditions reduced to 40, and a sweep across subsampling fractions is performed to show performance of BCLR on realistically sized data sets derived from the greater *E. coli* dataset. Bands indicate a 99% confidence interval constructed from the samples taken at each data fraction. Differences were significant (*p*-value < 0.01) for subsampling fractions 30% and below

While the *E. coli* dataset is informative from the standpoint of having many conditions to infer relationships from, the reality is that most experiments involve far smaller datasets, but will still use network inference in their analysis [4, 19, 20]. Accordingly, we looked at bootstrapping effects when initial dataset sizes were 40 conditions, which is closer to the size that might be obtained from a single experiment, or combining multiple smaller experiments for less-studied organisms. Examining the performance of BCLR on such datasets with varying subsampling fractions (Fig. 4) we found that CLR consistently outperformed BCLR. This indicates that, in contrast to a large initial dataset size (Fig. 1), there is no advantage to bootstrapping with smaller initial dataset sizes.

### Choice of inference method

**Fig. 5:**
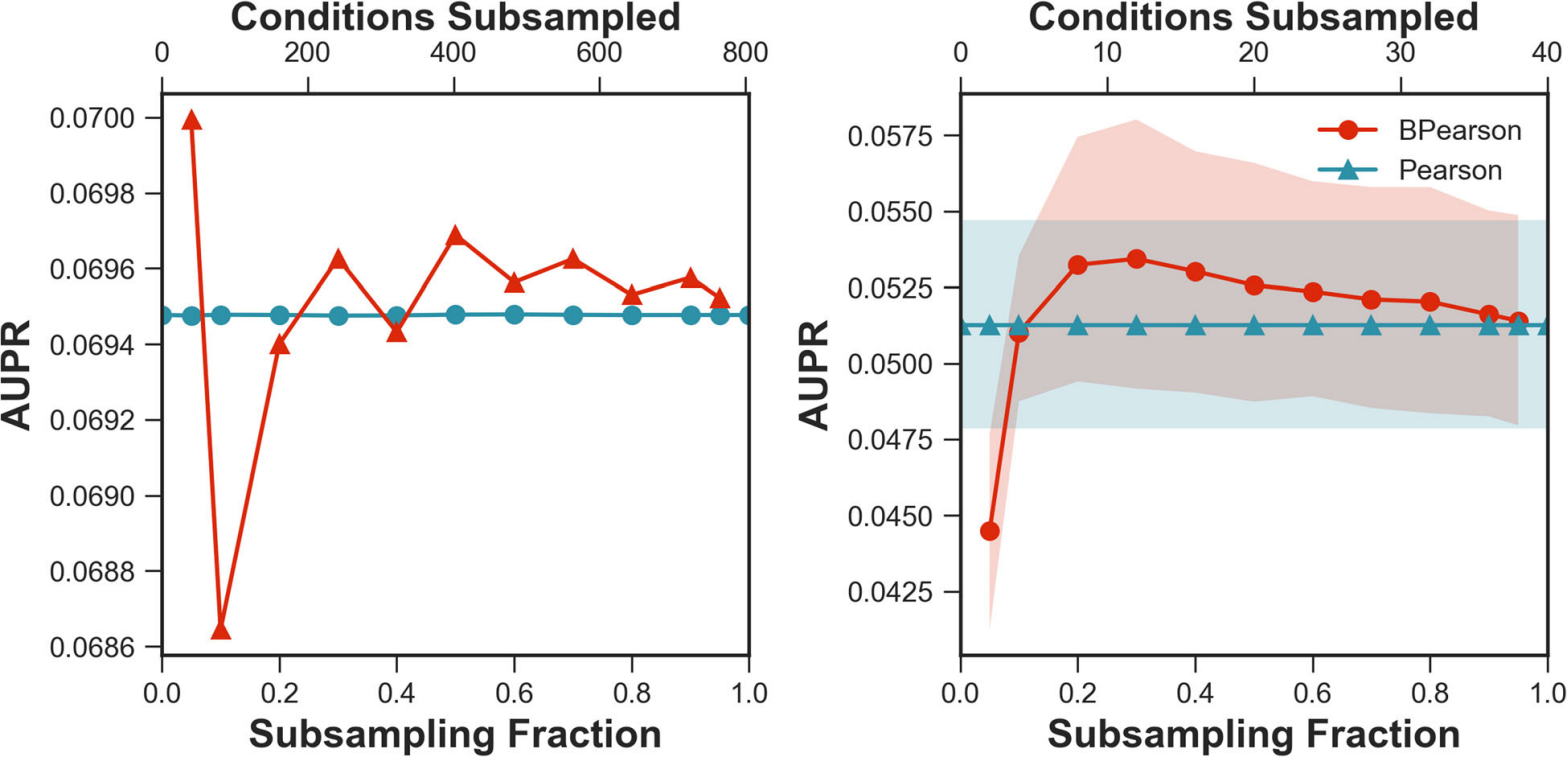
Bootstrapped Pearson correlation. Performance of Pearson and bootstrapped Pearson assessed on the full set of *E. coli* conditions (left) and on a realistically sized subset (right), both in terms of AUPR. In the right panel, bands indicate a 99% confidence interval constructed from the samples taken at each data fraction. The only significant difference (in the right panel, significance could not be assessed in the left due to sample size of 1) was for 5% subsampling fraction (*p*-value: 3.1E-3)

The CLR method is based on a mutual information metric, but many studies use simpler correlation metrics, like Pearson correlation, to predict associations between genes. We therefore used our bootstrapping approach, but applied it with Pearson correlation as the underlying inference method. We show performance of the method when applied to the full initial dataset and to reasonably sized (40 conditions) initial datasets in Fig. 5. The bootstrapped Pearson method shows improvements over the simple Pearson under most subsampling fractions, and interestingly, with the reasonably sized initial datasets, bootstrapped Pearson modestly outperforms CLR (simple or bootstrapped, see Fig. 4) with maximum AUPRs of 0.053 versus 0.050 for bootstrapped Pearson and bootstrapped CLR, respectively (*p*-value: 2.2E-4). This may not be surprising given that mutual information requires knowledge of the probability distribution, and so with smaller sample sizes the probability estimate might deviate from Gaussian distribution dramatically.

### Functional overlap

**Fig. 6:**
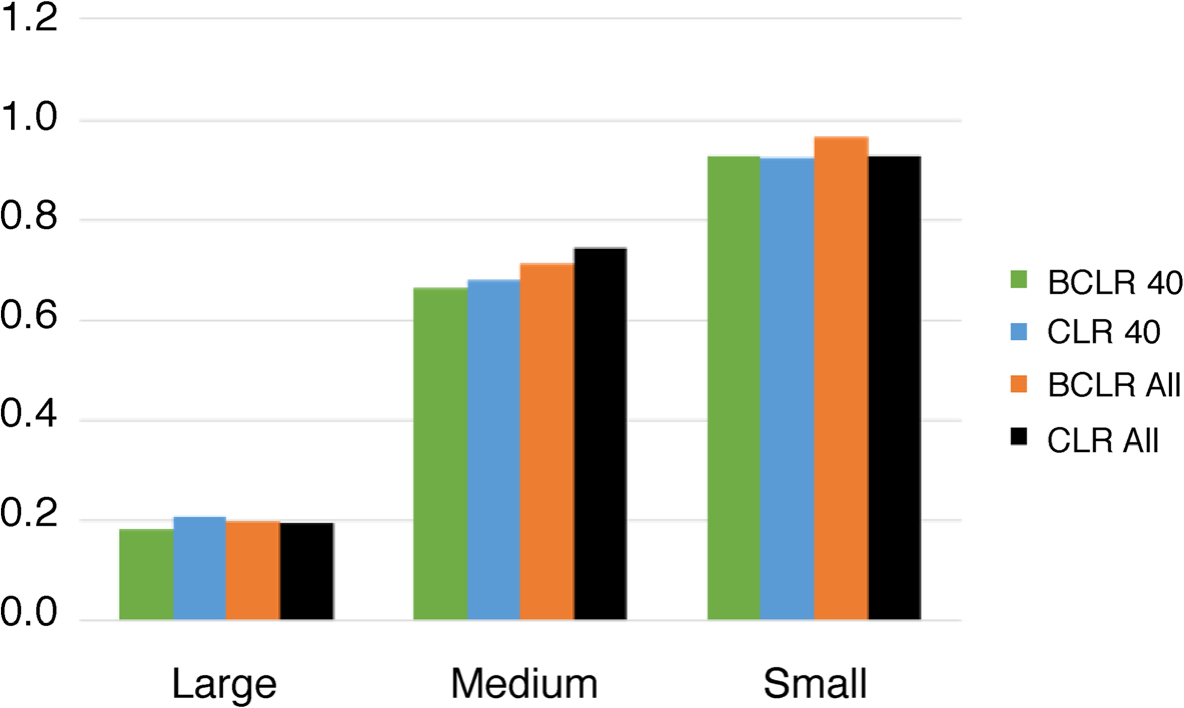
Functional enrichment analysis. The ratio of the functional enrichment overlap (number of edges connected annotated genes in the same category/number of edges connected any two annotated genes) is shown for *E. coli* networks made using BCLR and 40 datasets (green bars), using CLR and 40 datasets (blue bars), using BCLR and all datasets (orange bars) and using CLR and all datasets (black bars). Large (200000 edges), medium (10000 edges) and small (2000 edges) networks were examined

A primary motivation for the DREAM5 competition and for development of new network inference methods is to determine gene regulatory networks, which connect transcription factors with their regulated targets. Thus far, we have focused on this application using a set of known transcription factor-target pairs to assess performance, as done in DREAM5. However, network inference methods can also be used to determine functional overlap from transcriptional (or other) data across many conditions [4, 17, 21, 22]. Thus, we wanted to assess the ability of bootstrapped inference methods to infer edges between genes in the same pathways. Figure 6 shows the results of this analysis. This indicates that BCLR provides a slight advantage over CLR when the initial dataset size is small, but this advantage disappears when using a large initial dataset size.

## Discussion

### Computational overhead

Due to the additional computational overhead introduced by bootstrap iterations, applications must consider whether the marginal gains to stability and accuracy are justified. This is largely based on the size of the dataset, the inference algorithm used, and the availability of high-performance computing resources, and will vary on a case-by-case basis.

### Accuracy

Accuracy as evaluated by AUPR increased marginally over non-bootstrapped inference methods for certain dataset sizes and subsampling fractions. It must be maintained that these results hold for the limited datasets evaluated in this work, and with incomplete (silver) standards. Generalization to real-world datasets is assumed, but not guaranteed. Further, when performing network inference using unlabeled datasets, accuracy cannot be assessed. We can only employ the best-performing inference method, as assessed with known interactions. We can, however, assess stability without labeled data, but its use as a proxy for accuracy may not be justified, as discussed in the next section.

### Stability as a proxy for accuracy

Stability, that is, resilience to changes in the underlying data, is not necessarily correlated with accuracy, despite a general inverse relationship between AUPR and MAE (see Additional file 3: Figures S2 and Additional file 4: Figure S3). BCLR at 5% subsampling showed greatest AUPR improvement where no conditions were removed, which corresponds to the only point where BCLR was less stable than CLR. Similarly, with smaller datasets (i.e. > 60% conditions removed), BCLR was more stable than CLR, but CLR resulted in higher-accuracy networks. That said, when between 10 and 60% of conditions were removed, BCLR exceeded CLR in both metrics (Fig. 3). But without prior knowledge of true interactions, and therefore no standard by which to calculate AUPR, a novel study cannot optimize by accuracy.

By pursuing stability as a proxy and making use of all conditions, CLR would be selected over BCLR, despite the improved accuracy of the latter. Thus, our recommendation would be to optimize for the most stable *bootstrapped* network. That is, (i) construct a parent network at each subsampling fraction, (ii) for each subsampling fraction, iteratively remove conditions and run BCLR, comparing each result to the respective parent network, (iii) select the subsampling fraction that results in the most stable condition-removal curve, and finally (iv) use the resulting subsampling fraction with all available data.

### Functional edge overlap

Although AUPR is a measure of accuracy that has been used in a number of studies, including this one, we wanted to also include a more general measurement of network accuracy in addition to AUPR. Rather than focus only on regulator-target pairs we took advantage of the abundant functional annotation information available for *E. coli* and determined how often each network inference method could link genes in the same functional category. This approach revealed several strategies for inferring accurate networks, use as much data as possible, smaller networks are more accurate than larger ones and BCLR has a slight advantage over CLR with small networks but not with large networks.

## Conclusions

This work explored the effects of feature bootstrap aggregation on the network inference algorithm in terms of stability and accuracy. With respect to stability, BCLR improved over CLR at recreating its parent network across condition set sizes for small subsampling fractions (e.g. 5%). Larger subsampling fractions had little effect on resulting networks, providing marginal improvements in stability. In terms of accuracy, BCLR demonstrated a noticeable improvement over CLR at low subsampling fractions and for cases in which the initial pool of conditions was sufficiently large. This is also supported by FEO analysis. In concert, these findings reveal several important considerations when using CLR (or BCLR) to infer gene regulatory networks in novel studies where true interactions are unknown.

## Methods

### Datasets

In order to evaluate the effects of data structure and variability on network inference we chose to utilize a large transcriptional compendium previously used for the DREAM5 network inference competition [3]. This included gene expression data from 805 perturbations of *Escherichia coli.* For this dataset, the underlying network is not completely known, but we considered the set of known transcription factor-target relationships used by the DREAM5 competition to be the ‘silver standard’ for evaluation. This silver standard provides a reasonable way to compare the performance of different network inference approaches. In this study we focus on the use of gene expression measurements on network inference and so refer to each individual expression set arising from an individual perturbation as a ‘condition’.

### Network inference

Networks were inferred using the context likelihood of relatedness (CLR) algorithm [18] or Pearson correlation coefficient. For CLR, default parameters were used, except for number of bins and order of the fitted splines, which were set to 10 and 3, respectively. For each possible interaction, CLR produces a z-score that corresponds to the significance of the interaction given its contextual neighborhood. These values were used as-generated in deciding the confidence of a given edge.

### Evaluation

Network generation methods (e.g. with different bootstrapping parameters) were evaluated against respective parent networks and the silver standard to assess stability and accuracy, respectively*.* We define a parent network as the network inferred by a particular algorithm (CLR, Pearson, or their bootstrapped variants) using the entire set of conditions. We define stability as the similarity, evaluated as mean absolute error (MAE), between a network inferred from a subset of conditions and the network inferred from the complete set of data (the parent network). Accuracy is defined as how well a network captures the set of known, experimentally defined transcription factor-target relationships.

The provided standards contain edge information for 152,280 of approximately 10 million possible interactions, 2,066 of which are positive. To account for this sparsity when comparing to generated networks, the standard was cast as a dense matrix wherein absent edges were encoded as “not a number” (NaN), effectively masking unknown edges in performance evaluations, a practice generally accepted in the literature [3]. It is worth noting that the generated standard is a directed graph (e.g. an edge between node A and node B does not necessarily guarantee an edge between node B and node A) whereas the networks generated by Pearson correlation and CLR are undirected graphs (i.e. edges between two nodes exist in both directions).

Stability was evaluated by MAE with the respective parent network. Lower values indicate greater similarity and, by extension and our definition, stability. Accuracy was evaluated by area under the precision-recall curve (AUPR). AUPR is parameterized by threshold selection (here, a z-score cutoff) of a given test network benchmarked against a standard. This enables performance assessment without the need for explicit threshold selection, a nontrivial decision with implications beyond the scope of this work. There are other acceptable metrics to assess network similarity—root mean squared error, Jaccard index, etc.—but AUPR was selected for its wide use in biological network inference applications, because thresholds need not be selected, and its better performance compared to other classifiers (e.g. receiver operating characteristic) when dealing with imbalanced datasets [23].

We also determined accuracy by examining how many edges in the network connected two genes that were in the same functional category. Functional information for *E. coli* genes was obtained from EcoCyc [24]. We use a metric termed the functional edge overlap (FEO) ratio, the number of edges linking annotated genes in the same functional category divided by the number of all edges linking annotated genes. If an edge links to a gene that has no functional annotation that edge is ignored for the FEO analysis. FEO ratios can range from 0 (no edges link annotated genes in the same category) to 1.0 (all edges linked annotated genes in the same category).

### Feature bootstrap aggregation

In light of the fact that varying the number of conditions in biological network inference can have substantial impact on the inferred network (Fig. 1), we explored use of bootstrap aggregating of the conditions with the goal of generating a *stable* network. That is, a network less sensitive to the presence or absence of certain conditions. Additionally, we hypothesized that bootstrap aggregation would result in improved network inference performance (i.e. accuracy).

We implemented condition bootstrap aggregation by randomly selecting a fraction of the full condition set without replacement, then running CLR on this subset to generate a *constituent* network. This process was repeated *n* times until convergence (*n* = 200; Additional file 2: Figure S1). Convergence was demonstrated by evaluating the addition of each constituent network after each iteration in terms of AUPR with the silver standard, as well as MAE with the consensus network from the previous iteration. Resulting constituent networks were aggregated by averaging their edge weights, yielding a consensus network. The bootstrap-aggregated version of CLR is henceforth referred to as BCLR. Note also that we implemented a bootstrap-aggregated variant of Pearson’s correlation method for comparison purposes, but this algorithm was not the primary focus of this work.

### Subsampling fraction

Assuming CLR-specific parameters do not change, bootstrap aggregation introduces two additional parameters: number of bootstrap iterations and subsampling fraction. Number of iterations is chosen to ensure convergence (Additional file 2: Figure S1), so ultimately only subsampling fraction introduces additional complexity. A sweep of subsampling fractions was performed to assess its effect on performance in terms of accuracy only, as each network is itself the associated parent, barring stability assessment (Fig. 2). This was achieved by running BCLR with subsampling fractions ranging from 0.01 to 1.0.

### Limiting input conditions

Because we are assessing these methods on a very large dataset of transcriptional data from *E. coli* (> 800), we were also interested to see the effects of limiting the number of conditions considered to levels that would be more reasonable to expect for other organisms that have not been studied as extensively. We therefore compared performance of the methods (CLR and BCLR) on input data limited to randomly selected subsets of the initial data, from 10 to 90% of initial data. Because there is variability associated with which specific conditions are removed, this process was repeated 10 times for each fraction removed, from which a mean and confidence interval was constructed for the relevant plots. For information on how the confidence intervals were constructed, see Additional file 1: Supplemental Results.

### Significance testing

Differences in mean AUPR and/or MAE were tested for significance using a two-sided T-test with the null hypothesis that sample means are equal. Unequal population variances were assumed when conducting the T-test, as sample variances were observed to differ between results for each algorithm (CLR, Pearson) and their bootstrapped variants (BCLR, BPearson). *P*-values are reported directly.

## Additional files

Additional file 1: Supplemental Information for Improving network inference and functional module identification using resampling methods. (DOCX 16 kb)

Additional file 2: **Figure S1.** Figure for bootstrap aggregation convergence results. (PNG 44 kb)

Additional file 3: **Figure S2.** Figure showing AUPR versus MAE for inferred networks. (PNG 72 kb)

Additional file 4: **Figure S3.** Figure showing effect of varying number of conditions on stability and accuracy. (PNG 216 kb)

## Abbreviations

(B)C3NET: (Bootstrapped) Conservative Causal Core (C3) Network
(B)CLR: (Bootstrapped) Context Likelihood of Relatedness
ARCANE: Algorithm for the Reconstruction of Accurate Cellular Networks
AUPR: Area under the precision-recall curve
DREAM: Dialogue for Reverse Engineering Assessments and Methods
FEO: Functional edge overlap
GENIE3: GEne Network Inference with Ensemble of trees
MAE: Mean absolute error
NaN: Not a number
RLowPC: Relevance Low order Partial Correlation
