# Supplementary material for: Improving network inference algorithms using resampling methods
Source: BMC Bioinformatics. 2018 Oct 12;19:376. doi: 10.1186/s12859-018-2402-0 (PMC6186128; doi:10.1186/s12859-018-2402-0)
Supplement: Supplementary file 1 — Supplemental Information for Improving network inference and functional module identification using resampling methods. (DOCX 16 kb) [file 12859_2018_2402_MOESM1_ESM.docx]

Supplemental Information for Improving network inference and functional module identification using resampling methods

*Sean M. Colby^1^, Ryan S. McClure^1^, Christopher C. Overall ^1†^, Ryan S. Renslow^1^, Jason E. McDermott^1^**

^1^ Earth and Biological Sciences Directorate, Pacific Northwest National Laboratory, Richland, Washington, USA

^†^ Current location: Center for Brain Immunology and Glia, University of Virginia, Charlottesville, Virginia, USA

* Corresponding author: Jason E. McDermott (jason.mcdermott@pnnl.gov)

Author emails: sean.colby@pnnl.gov, ryan.mcclure@pnnl.gov, co4p@eservices.virginia.edu, ryan.renslow@pnnl.gov, jason.mcdermott@pnnl.gov

RESULTS

**Convergence.** To examine the effect of increasing the number of bootstrap iterations on resulting network stability we compared the consensus network of each bootstrap iteration with the consensus network from the previous iteration by MAE. A low MAE means that the network agrees well with the previous iteration, and has thus converged to a stable answer. Supplemental Figure 1 shows convergence in MAE of BCLR using 5% subsampling fraction. Note that we consider the result to have converged after 200 iterations, but the error is nonzero, meaning some variability is introduced by bootstrapping conditions.

**Larger Subsampling Fraction.** While the most improvement was observed with 5% subsampling of the entire dataset, we wanted to explore the effects of a larger subsampling fraction, paying particular attention to data fractions where BCLR performed poorly with 5% subsampling (i.e. <40% remaining conditions). Results indicate that BCLR with 80% subsampling fraction maintained approximately the same level of stability as CLR, regardless of the number of input conditions. With respect to resulting network accuracy, BCLR was largely indistinguishable from CLR, as any differences were within the noise associated with sampling from the pool of conditions, per Supplemental Figure 2.

**Confidence Interval Construction.** For analysis that introduced variability due to condition subset selection, the network inference process was repeated 10 times, each of which with a different random subset of conditions. Confidence intervals of the mean were constructed from each set of 10 samples and expressed as bands in relevant figures. Intervals were calculated by non-parametrically resampling the data 5000 times to obtain an estimate of the bootstrap distribution, as detailed in [1].

REFERENCES

1. Carpenter J, Bithell J: **Bootstrap con" dence intervals: when, which, what? A practical guide for medical statisticians**. 2000.

FIGURE LEGENDS

**Supplemental Figure 1**. Bootstrap aggregation converges by 200 iterations in terms of MAE, where the error is calculated between the consensus network of the current and previous iteration, respectively.

**Supplemental Figure 2.** Plotting AUPR for MAE for all inferred networks in this study shows an inverse relationship, though this trend is likely confounded by the relationship between MAE and data fraction removed: as more data is removed, MAE increases and AUPR decreases.

**Supplemental Figure 3.** Subsampling fraction was held constant (80%) and number of conditions were varied to demonstrate stability in terms of MAE (left) and effect on accuracy in terms of AUPR (right). Bands indicate a 99% confidence interval constructed from the samples taken at each data fraction. For MAE, significant differences were observed for 0.05 and 1.0 data fraction remaining (p-values 5.6E-04 and 9.1E-06, respectively). For AUPR, the only significant difference was observed when no data was removed (data fraction remaining: 1.0, p-value: 2.6E-06).
