# Supplementary figures and images for: Improving network inference algorithms using resampling methods
Source: BMC Bioinformatics. 2018 Oct 12;19:376. doi: 10.1186/s12859-018-2402-0 (PMC6186128; doi:10.1186/s12859-018-2402-0)

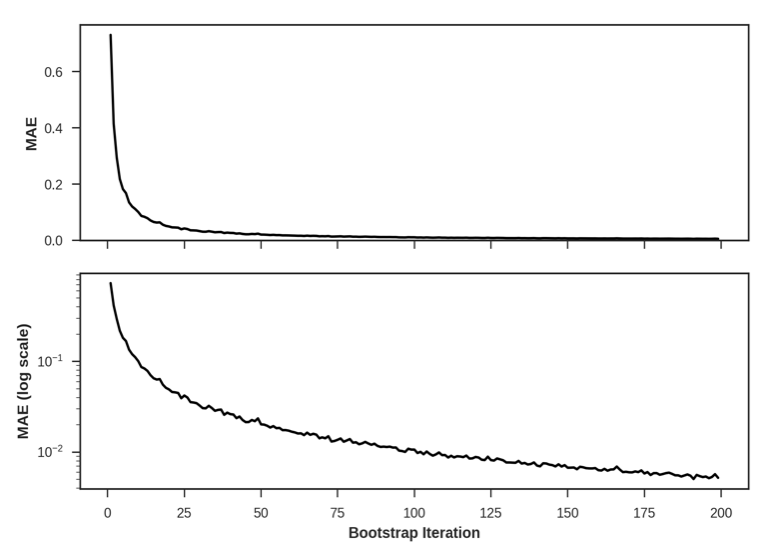

Supplement: Supplementary file 2 — Figure S1. Figure for bootstrap aggregation convergence results. (PNG 44 kb) [file 12859_2018_2402_MOESM2_ESM.png]

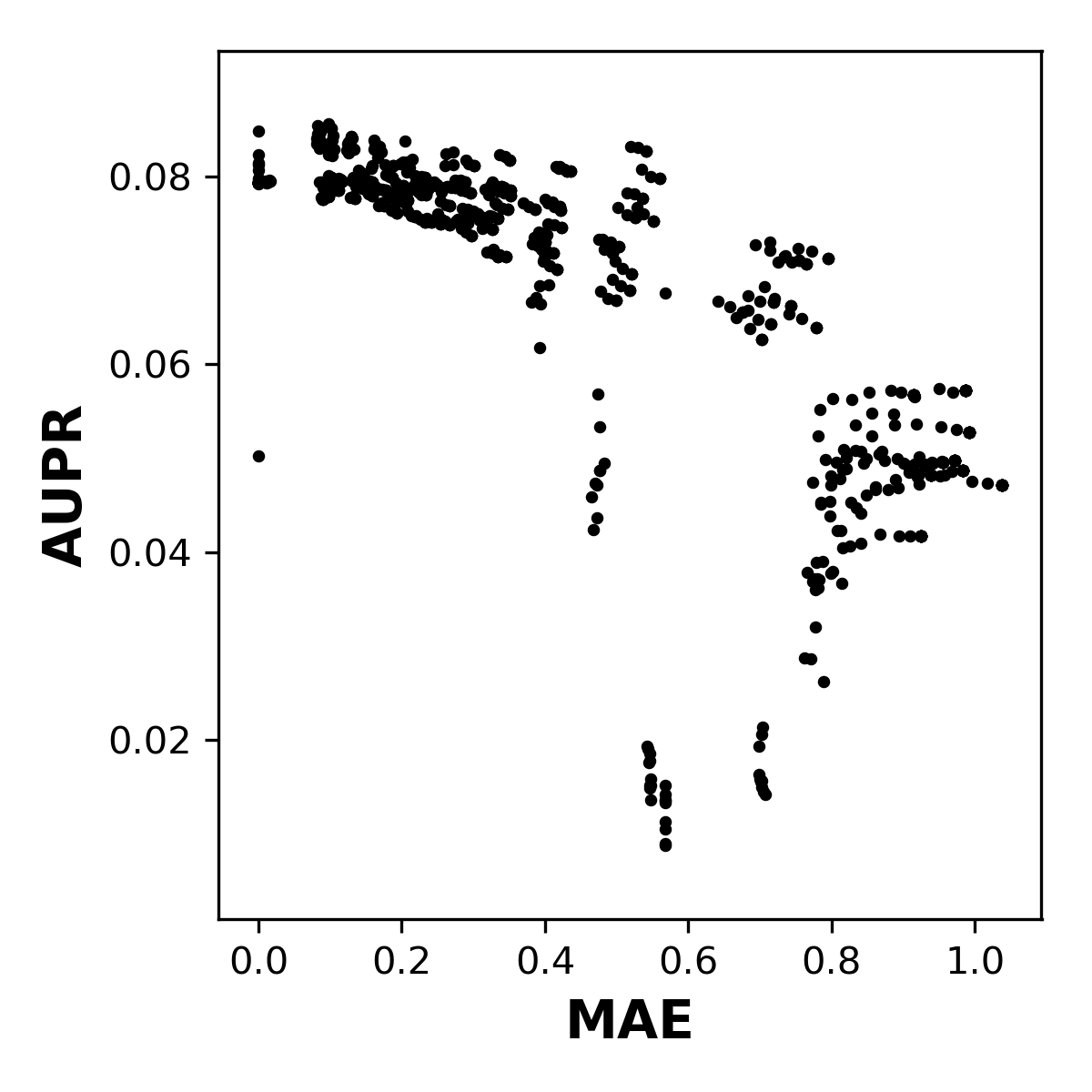

Supplement: Supplementary file 3 — Figure S2. Figure showing AUPR versus MAE for inferred networks. (PNG 72 kb) [file 12859_2018_2402_MOESM3_ESM.png]

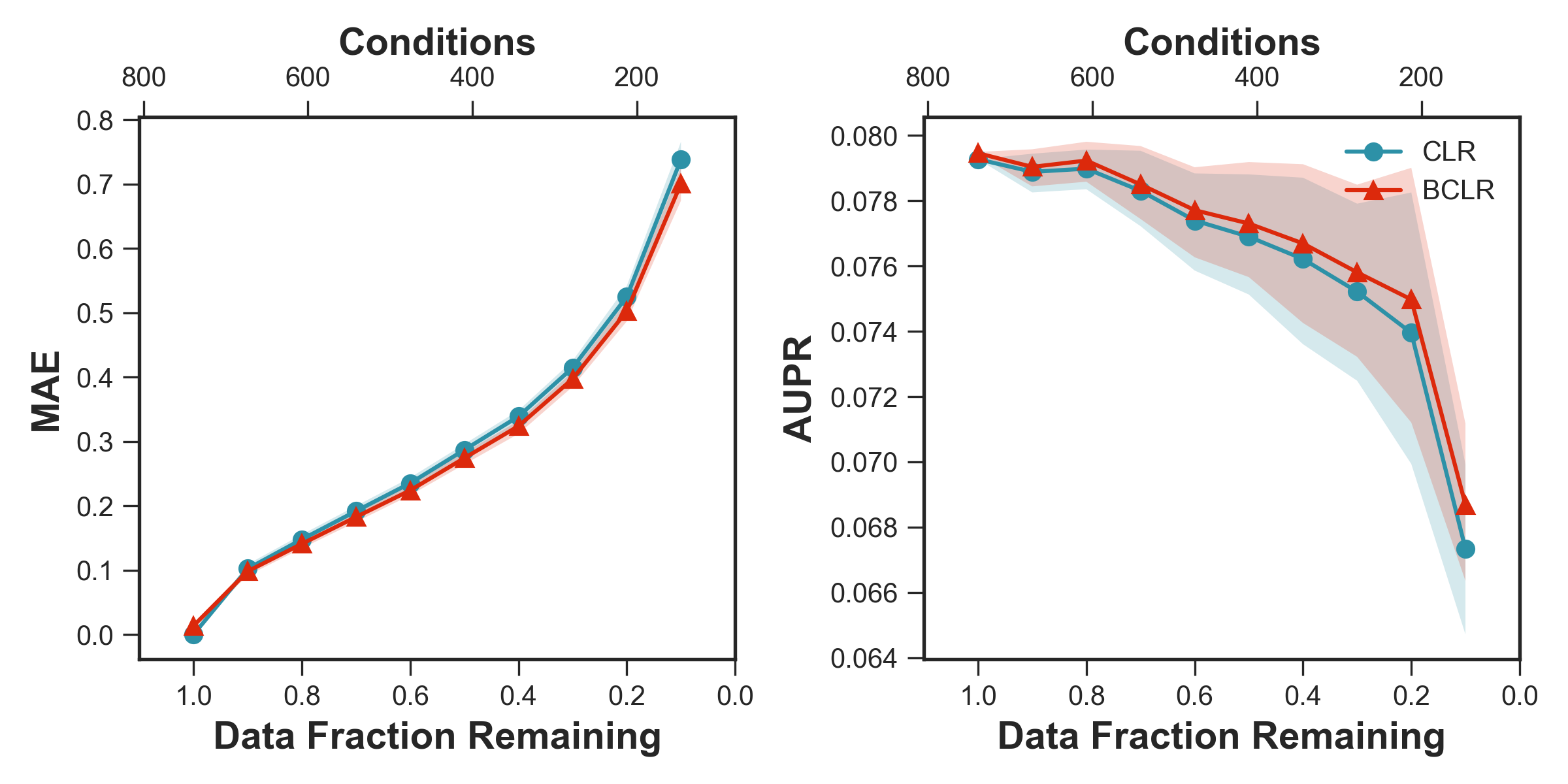

Supplement: Supplementary file 4 — Figure S3. Figure showing effect of varying number of conditions on stability and accuracy. (PNG 216 kb) [file 12859_2018_2402_MOESM4_ESM.png]
